# Comparative analysis of BPA and HQ toxic impacts on human erythrocytes, protective effect mechanism of tannins (*Rhus typhina*)

**DOI:** 10.1007/s11356-017-0520-2

**Published:** 2017-10-29

**Authors:** Ewa Olchowik-Grabarek, Katerina Makarova, Saidmukhtar Mavlyanov, Nodira Abdullajanova, Maria Zamaraeva

**Affiliations:** 1 Department of Biophysics, University of Bialystok, Ciolkowskiego 1J, 15-245 Bialystok, Poland; 2 Department of Physical Chemistry, Faculty of Pharmacy, The Medical University of Warsaw, Banacha 1, 02-097 Warsaw, Poland; 3 Institute of Bioorganic Chemistry, Academy of Science of Uzbekistan, Abdullaev 83, Tashkent, Uzbekistan 100125

**Keywords:** Tannins, Bisphenol A, Hydroquinone, Membrane fluidity, EPR, Erythrocytes, Hemolysis, Methemoglobin, Glutathione, Rhus

## Abstract

Several studies reported that bisphenol A (BPA) and its metabolite hydroquinone (HQ) have adverse effects on human and animal health. In this work, a comparative study of influence of the BPA and HQ, environment pollutants, on human erythrocytes was carried out. It was shown that BPA and HQ to varying extents caused oxidative damage in human erythrocytes: hemolysis, decreased GSH level, and methemoglobin formation. It was demonstrated that hydrolysable tannins 3,6-bis-*O*-di-*O*-galloyl-1,2,4-tri-*O*-galloyl-β-d-glucose (C_55_H_40_O_34_) and 1,2,3,4,6-penta-*O*-galloyl-β-d-glucose (C_41_H_32_O_26_) (PGG) isolated from the *Rhus typhina* L. leaves in the range of 1–50 μM concentrations inhibited hemolysis and methemoglobin formation and also increased intracellular reduced glutathione in erythrocytes treated with BPA or HQ. It was revealed by electron paramagnetic resonance (EPR) using 5-doxyl-stearic acid (5-DS) that C_55_H_40_O_34_ and C_41_H_32_O_26_ increased the rigidity of erythrocyte membranes at the depth of 5th carbon atom of the fatty acid hydrocarbon chain. Taken together, these results allow to conclude that tannins from the *Rhus typhina* L. leaves protect erythrocytes from oxidative stress caused by BPA or HQ both due to their antioxidant activity as well as their interaction with the erythrocyte membrane components.

## Introduction

Bisphenol A (BPA) is one of the components of epoxy resins and plastics that are used to manufacture a great variety of different goods widely used in everyday life, such as: bottles for water and various beverages, baby food bottles, food packaging containers, compact discs, etc. Moreover, BPA is also a component of items commonly used in medicine, e.g. contact lenses, orthodontic materials and instrumentation (Welshons et al. 2006; Konieczna et al. 2015; Halimi et al. 2016).

Throughout the whole BPA history much evidence has been accumulated for its toxic effects on human health (Metz 2016). BPA is readily released into the environment, particularly upon heating, which opens up an additional way for its adverse effects on the human body (Konieczna et al. 2015). BPA was detected in aquatic environment, soil, air, food, and the human body (Huang et al. 2012; Suzuki et al. 2004).

BPA is one of the most extensively studied endocrine disruptors in terms of its long-term biologic effects. Its chemical structure is similar to the structure of synthetic non-steroid estrogen diethylstilbestrol which exerts a harmful effect on the reproductive system in women (Welshons et al. 2006).

It was found that BPA induced the development of breast and prostate cancers, polycystic ovary syndrome (Deb et al. 2016; Kandaraki et al. 2011), as well as disturbances in spermatogenesis and fertility (Singh et al. 2015). BPA also has an embryotoxic effect, which manifests itself in impairment of the development of genitals in adult animals and the increase of their cancer susceptibility (Prins et al. 2008).

However, apart from the estrogenic effect, according to available data bisphenol compounds disturb the prooxidant/antioxidant balance in cells of various tissues, which results in oxidative damage of their essential components such as lipids, proteins, and nucleic acids, finally leading to impairment of organ functional activity (Babu et al. 2013). The BPA was also shown to exert a hepatotoxic effect, inducing oxidative stress in liver cells (Kourouma et al. 2015). The in vivo experiments revealed that the cardiotoxic effect in adult male rats was mediated by oxidative stress (Aboul Ezz et al. 2015). There is evidence that BPA also induced oxidative damage in rat testis (Wang et al. 2016). The in vivo experiments demonstrated that the BPA-provoked change in spermatozoon functional activity was a result of its prooxidative effect on mitochondria (Singh et al. 2015; Barbonetti et al. 2016).

Some works showed a cytotoxic action of BPA on human erythrocytes that are among the first targets of toxic effect of hydrophobic pollutants transferred by blood (Macczak et al. 2015, 2016; Suthar et al. 2014). Not only the BPA itself is toxic but also the products of its degradation that are formed as a result of microbial degradation and during the wastewater treatment by different methods (Kolvenbach et al. 2007; Yang 2015).

One of them is hydroquinone (HQ) which is compound of monooxygenase transformation of BPA by cytochrom related enzymes (Sasaki et al. 2005; Atkinson and Roy 1995; Kolvenbach et al. 2007). Strong ability of HQ to induce apoptosis was demonstrated for human embryonic kidney cells (HEK293) (Shen et al. 2003). HQ was found to induce genotoxicity effect in human lymphocytes and human lung alveolar epithelial cells (A549) via ROS formation (Peng et al. 2012, 2013).

It was shown that HQ exhibits a higher toxicity compared with BPA in relation to human promyelocytic leukemia (HL-60) and human oral squamous cell carcinoma (HSC-2) cells (Terasaka et al. 2005). However, zebrafish embryo toxicity test showed that HQ was much less toxic then BPA (Makarova et al. 2016).

It is well known that plant polyphenols, including tannins, are powerful protectors against pathological changes caused by prooxidants of different nature, including the pollutants (Chen et al. 2016; Shafiee et al. 2003; Ciftci et al. 2012). The mains mechanisms of the protective effect of polyphenols is based on the reaction with free radicals, chelation of transition metals and increased expression of antioxidant enzymes (Koleckar et al. 2008; Fraga and Oteiza 2011; Hagerman et al. 1998).

It should be noted that in the literature, there are very few papers describing the protective effect of antioxidants against the cytotoxicity of BPA. It was shown earlier that antioxidants such as catechins of green and black tea and quercetin exhibit the antihemolytic effect (Suthar et al. 2014; Verma and Sangai 2009), whereas α-tocopherol and α-lipoic acid inhibit oxidative damage in liver and ovarian tissue of rats induced by BPA (Avci et al. 2016). However, it should be noted that there is some specificity in the action of antioxidants against oxidative stress induced by BPA. For example, vitamin C did not protect fish hepatocytes from oxidative damage caused by BPA but on the contrary aggravated its negative effects (Kaya and Kaptaner 2016).

In our earlier publications, we showed that tannins isolated from sumac leaves (*Rhus typhina* L.) exhibit a high antiradical acivity in relation to ROS and protected the erythrocytes against oxidative stress caused by HClO, ONOO^−^, and tBuOOH, but to a various degree depending on the type of oxidant (Olchowik et al. 2012; Olchowik-Grabarek et al. 2017).

**Fig. 1:**
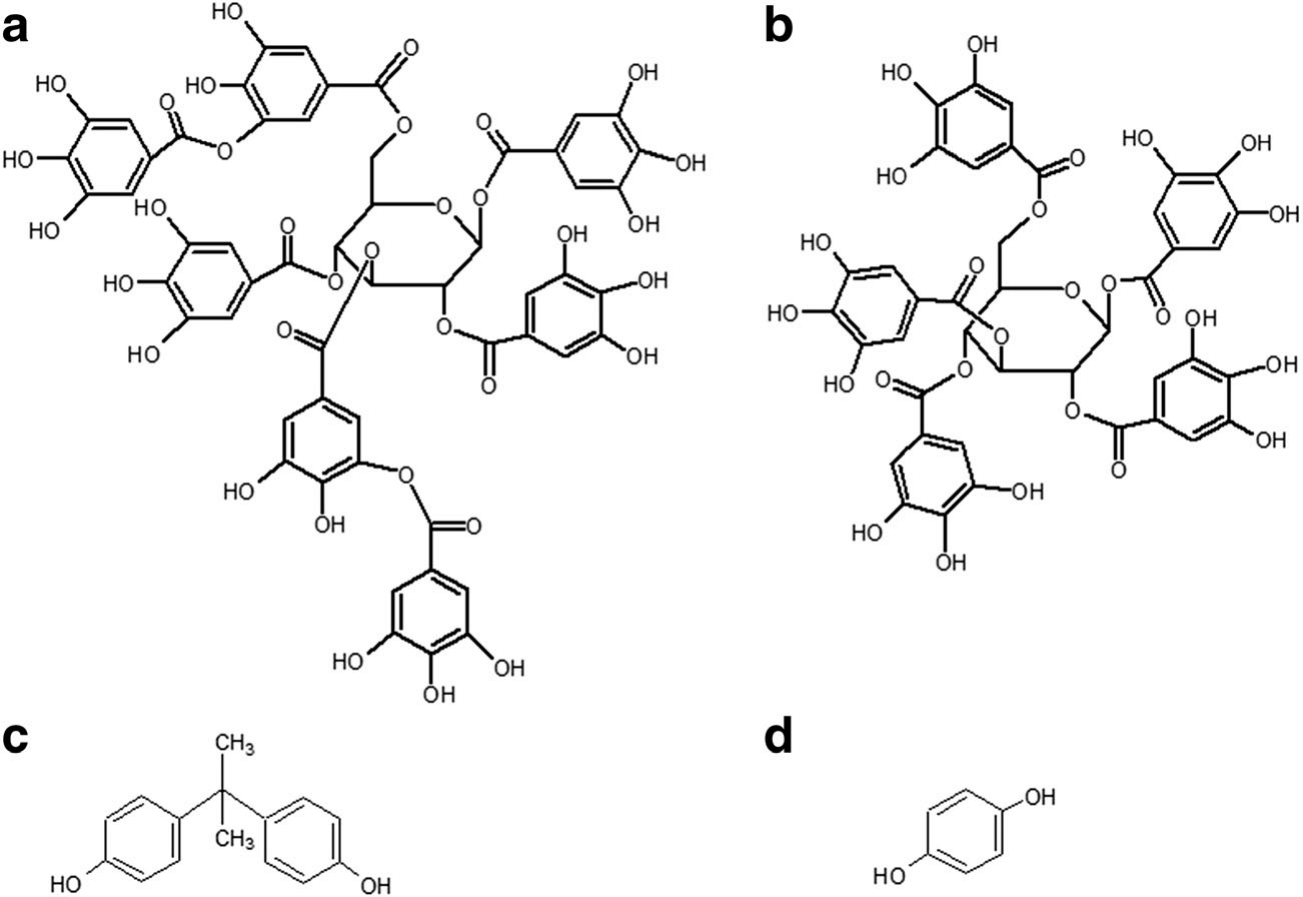
Chemical structure of 3,6-bis-*O*-di-*O*-galloyl-1,2,4-tri-*O*-galloyl-β-d-glucose (C_55_H_40_O_34_) (**a**), 1,2,3,4,6-penta-*O*-galloyl-β-d-glucose (C_41_H_32_O_26_) (PGG) (**b**), bisphenol A (BPA) (**c**), and hydroquinone (HQ) (**d**)

In this work, we carried out a comparative study of influence of the BPA and HQ on human erythrocytes and also estimated the capability of two hydrolysable tannins 3,6-bis-*O*-di-*O*-galloyl-1,2,4-tri-*O*-galloyl-β-d-glucose (C_55_H_40_O_34_) and 1,2,3,4,6-penta-*O*-galloyl-β-d-glucose (C_41_H_32_O_26_) (PGG) (Fig. 1) isolated from *Rhus typhina* L. leaves to protect cells against oxidative damage caused by these toxicants.

## Materials and methods

Anticoagulated blood samples were kindly provided by the Regional Blood Center in Bialystok. The study is approved by the ethics committee of the Medical University of Bialystok (R-I-002/77/2015).

## Chemicals

BPA (2,2-bis(4-hydroxyphenyl)propane), 5,5′-dithiobis(2-nitrobenzoic acid (DTNB, Ellman’s reagent), 5-doxylstearic acid (5-DS), and HQ were from Sigma-Aldrich. All other reagents were purchased from POCH (Poland).

## Plant material

The leaves of *Rhus typhina* L. were collected in Tashkent environs (Uzbekistan) and taxonomically identified in the Institute of Botanic of Academy of Science. The compounds: 3,6-bis-*O*-di-*O*-galloyl-1,2,4-tri-*O*-galloyl-β-d-glucose (C_55_H_40_O_34_) and 1,2,3,4,6-penta-*O*-galloyl-β-d-glucose (C_41_H_32_O_26_) (PGG) were isolated in the Institute of Bioorganic Chemistry of the Uzbekistan Academy of Sciences (Islambekov et al. 1994).

## Isolation of erythrocytes

Human blood was collected in tubes containing 18 mg EDTA/10 mL as an anticoagulant. Blood was centrifuged (600×*g*, 15 min, 4 °C). Erythrocytes were washed twice with 0.9% NaCl and then 1% suspension was prepared.

## Hemoglobin oxidation

One milliliter of 1% suspension of erythrocytes (PBS, pH 7.4) was incubated with C_55_H_40_O_34_ or C_41_H_32_O_26_ at 37 °C for 30 min. Next, the suspension was incubated for 4 h with 200 μg/mL BPA or HQ. After incubation, the erythrocytes were lysed with distilled water (1:4 ratio) and the samples were centrifuged (5000×*g*, 15 min, 4 °C). The percent of metHb was calculated by the formula (Macczak et al. 2015):

$$ \mathrm{metHb}\ \left(\%\right)=\frac{\left({A}_{630}-{A}_{700}\right)}{\left({A}_{100\%630}-{A}_{100\%700}\right)}\times 100\% $$

where metHb (%) is the percent of metHb; *A*_630_ and *A*_700_ is the absorbance of the sample at 630 and 700 nm; and *A*_100%630_ and *A*_100%700_ are absorbances of the sample at 630 and 700 nm treated with potassium ferricyanide. The results are expressed as percent of metHb formation in relation to completely oxidative Hb taken as 100%.

## Determination of GSH content

One milliliter of 1% suspension of erythrocytes (PBS, pH 7.4) was incubated with C_55_H_40_O_34_ or C_41_H_32_O_26_ at 37 °C for 30 min. Next, the suspension was incubated for 4 h with 200 μg/mL BPA or 50 μg/mL HQ and then 0.2 mL of 25% trichloroacetic acid was added. The samples were centrifuged (400×*g*, 10 min). To 0.5 mL of the supernatant, 0.5 mL 0.5 M phosphate buffer (pH 7.8) and 0.05 mL Ellman’s reagent (5 mM) were added. After 30 min, the samples were monitored spectrophotometrically at 414 nm (Olchowik et al. 2012). PBS solution with Ellman’s reagent was used as a blind sample. The GSH content in treated erythrocytes was presented as percent in relation to control erythrocytes taken as 100%.

## Measurement of erythrocyte hemolysis

One milliliter of 1% suspension of erythrocytes (PBS, pH 7.4) was incubated with C_55_H_40_O_34_ or C_41_H_32_O_26_ at 37 °C for 30 min. Next, the suspension was incubated for 24 h with 200 μg/mL BPA or HQ. After incubation, 4 mL of buffer (150 mM NaCl, 10 mM Tris-HCl, pH 7.4) was added to the samples and centrifuged (400×*g*, 15 min). The absorbance of hemoglobin in the supernatant was measured at 540 nm. The results are presented as percent of hemolysis in relation to hemolyzed erythrocytes taken as 100%.

## Analysis of erythrocyte membrane fluidity (a spin label study)

The fluidity of the erythrocyte membranes was measured using 5-doxyl-stearic acid (5-DS) by EPR method. Of 2% suspension, 0.1 mL of erythrocytes (PBS, pH 7.4) was labeled with 100 μM 5-DS and then incubated with C_55_H_40_O_34_ or C_41_H_32_O_26_ at 37 °C for 1 h. EPR spectra were recorded at room temperature (21 ± 1 °C) using an Adani CMS 8400 spectrometer, operating at a microwave frequency of 9.4 GHz. The instrumental settings were as follows: center field, 3360 G; scan range, 150 G; and modulation amplitude, 1 G.

The order parameter (*S*) was calculated by the equation:

$$ S=\frac{A_{\parallel }-{A}_{\perp }}{A_{ZZ}-\frac{1}{2}\left({A}_{XX}+{A}_{YY}\right)}\times \frac{a}{a^{\prime }} $$

The values of *a*′ were calculated by the formula:

$$ {a}^{\prime }=\frac{1}{3}\left({A}_{\parallel }+2{A}_{\perp}\right) $$

The value of *a* was calculated by the formula:

$$ a=\frac{1}{3}\left({A}_{XX}+{A}_{YY}+{A}_{ZZ}\right) $$

where *S* is order parameter; *a*′ is the isotropic hyperfine constant for nitroxide in a membrane; *a* is the isotropic hyperfine constant for nitroxide in a crystal; *A*_∥_ and *A*_⊥_ are the indicators measured directly in an experiment (Fig. 5); *A*_*XX*_, *A*_*YY*_ , and *A*_*ZZ*_ are the main values of the superfine splitting tensor, obtained on monocrystals in the absence of any molecular motion and equal, respectively, to 6.1, 6.1 and 32.4 G.

The rotational correlation time (*τ*_c_) was calculated by the equation:

$$ {\tau}_C=\frac{1}{2}k{w}_0\left(\sqrt{\frac{h_0}{h_{+1}}}+\sqrt{\frac{h_0}{h_{-1}}}-2\right) $$

where *τ*_c_ is the rotation correlation time; *k* is the constant equal to 1.19^.^10^−9^ s; *w*_0_ is the mid-field line width; *h*_0_ is the mid-field line height; *h*_+1_ is the low-field line height; and *h*_−1_ is the high-field line height (Hordienko et al. 1994; Nowak and Nedoszytko 2005). The results are presented as rotational correlation time (ns) and as order parameter in relation to control probe taken as 1.

## Statistical analysis

The results are presented as mean ± SE. The level of significance was analyzed using one-way ANOVA test. *p* < 0.05 and below was accepted as statistically significant. Statistical analysis was performed using Origin 8.5.1 (Microcal Software Inc., Northampton, MA) software.

## Results

### Effect of BPA and HQ and co-administration of tannins on the level of metHB, GSH, and erythrocyte hemolysis

**Fig. 2:**
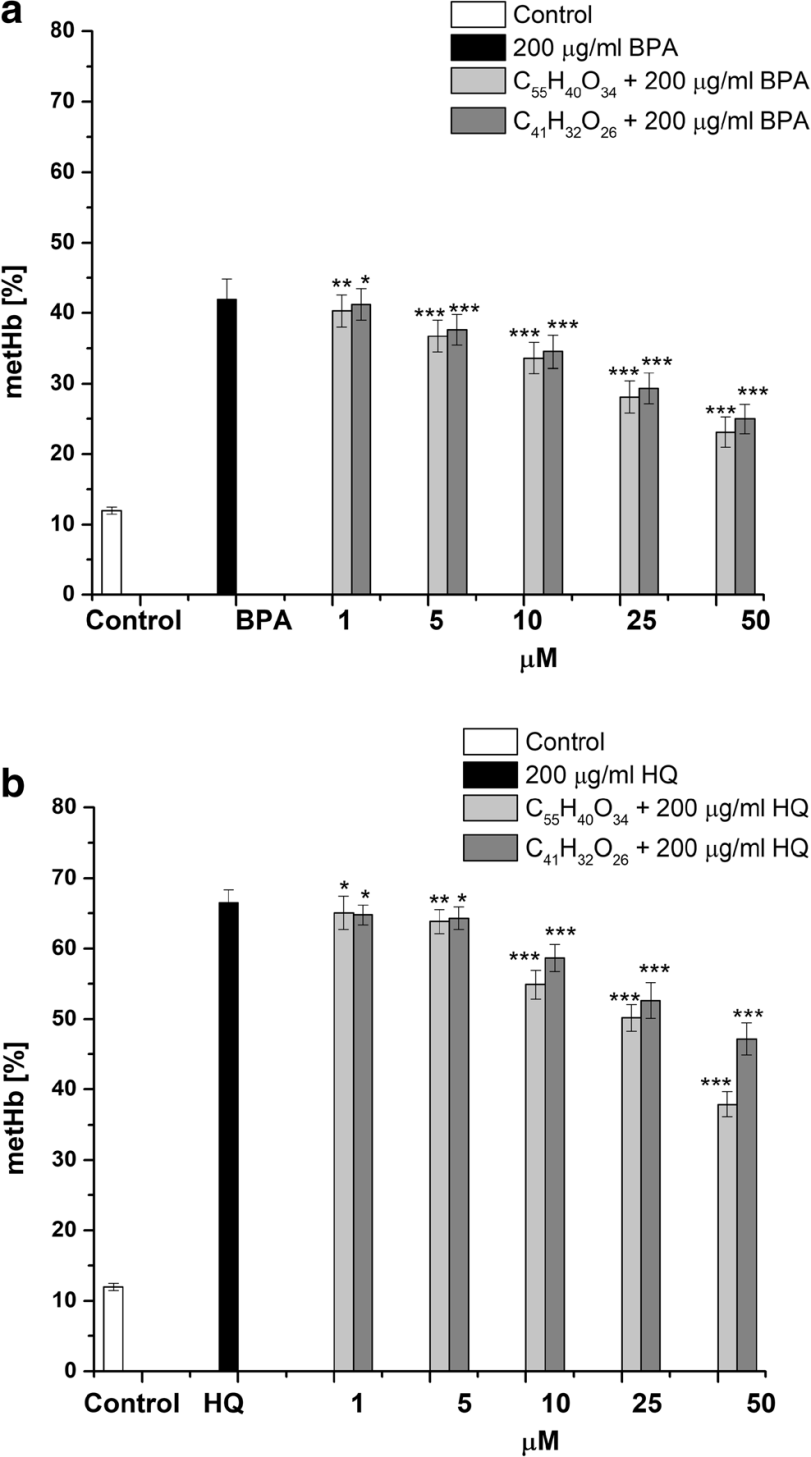
Protective effect of C_55_H_40_O_34_ and C_41_H_32_O_26_ against formation of methemoglobin in erythrocytes induced by 200 μg/mL BPA (**a**) or HQ (**b**). The data presented as the means ± SE (*n* = 10). The effects of compounds were statistically significant according to one-way ANOVA test (*p < 0.05; **p < 0.01; ***p < 0.001)

In this study, we observed that the incubation of the erythrocytes with 200 μg/mL BPA or HQ gave a statistically significant increase of metHb level up to 42.06 ± 2.81% (*p* < 0.001) and 66.52 ± 1.83% (*p* < 0.001) versus control samples (12.21 ± 0.51%) (Fig. 2). HQ induced a 24.46 ± 2.11% (*p* < 0.001) increase in the production of metHb in comparison with BPA.

Pretreatment erythrocytes with C_55_H_40_O_34_ and C_41_H_32_O_26_ lead to dose-dependent (1–50 μM) decrease of metHb formation induced by BPA as well by HQ (Fig. 2a, b). Based on the results obtained, concentration of studied tannin causing 25% reduction metHb formation (EC_25_) was calculated by the method of linear regression. These data are presented in Table 1. According to these data, the protective activity of C_55_H_40_O_34_ in case of BPA was 1.61 ± 0.11 (*p* < 0.001) times as high as HQ. A similar effect was obtained for the C_41_H_32_O_26_, and the value for BPA was 1.55 ± 0.25 (*p* < 0.001) time as high as that for HQ.

**Table 1:** Protective effect of tannins, expressed as EC_25_, against erythrocytes oxidative damage induced by BPA or HQ

	EC_25_ (μM)			
	BPA		HQ	
	C_55_H_40_O_34_	C_41_H_32_O_26_	C_55_H_40_O_34_	C_41_H_32_O_26_
metHb	17.01 ± 1.34	25.61 ± 2.16	27.42 ± 2.04	39.81 ± 1.14
GSH	17.54 ± 1.47	24.82 ± 1.99	9.72 ± 0.44	18.61 ± 1.08
Hemolysis	23.66 ± 2.03	36.82 ± 2.31	12.04 ± 1.02	15.35 ± 1.11

At all tested concentrations, C_55_H_40_O_34_ was found to more strongly prevent the formation of metHb under the influence of BPA or HQ, as compared with C_41_H_32_O_26_. Comparison of EC_25_ values showed that effect C_55_H_40_O_34_ was 1.5 ± 0.17 (*p* < 0.001) and 1.45 ± 0.22 (*p* < 0.001) times higher in comparison with C_41_H_32_O_26_ in case BPA and HQ, respectively.

**Fig. 3:**
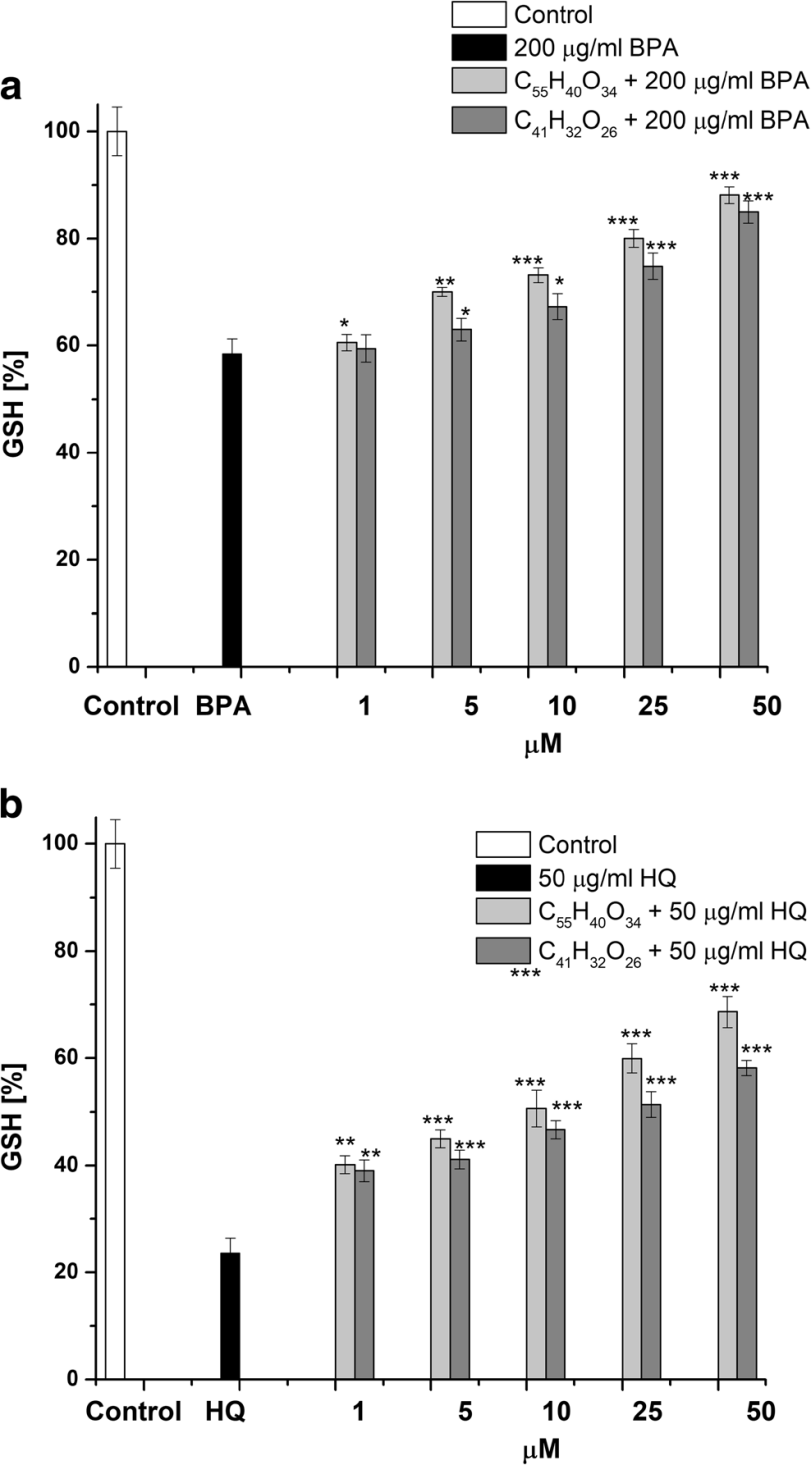
Sparing effect of C_55_H_40_O_34_ and C_41_H_32_O_26_ on GSH depletion in erythrocytes induced by 200 μg/mL BPA (**a**) or 50 μg/mL HQ (**b**). The data presented are the means ± SE (*n* = 10). The effects of compounds were statistically significant according to one-way ANOVA test (*p < 0.05; **p < 0.01; ***p < 0.001)

In other set of experiments, we showed that the exposure of erythrocytes to 200 μg/mL BPA or 50 μg/mL HQ caused a decrease in GSH content to 58.44 ± 2.81% (*p* < 0.001) and 23.58 ± 2.81% (*p* < 0.001), respectively, in comparison with control erythrocytes (100 ± 4.54%). As shown in Fig. 3, the presence of C_55_H_40_O_34_ and C_41_H_32_O_26_ in the concentration range of 5–50 μM together with 200 μg/mL BPA caused an increase of the GSH content. Similar protective effect of C_55_H_40_O_34_ and C_41_H_32_O_26_ (1–50 μM), we observed in case of erythrocytes in the presence of 50 μg/mL HQ. Analysis of the results indicates that values EC_25_ for C_55_H_40_O_34_ were 1.42 ± 0.28 (*p* < 0.05) and 1.91 ± 0.43 (*p* < 0.01) times higher in relation to C_41_H_32_O_26_ for content of GSH in erythrocytes exposed to BPA and HQ, respectively (Table 1).

Comparison of the tannins effects in two toxic models showed that C_55_H_40_O_34_ and C_41_H_32_O_26_ were 1.8 ± 0.35 (*p* < 0.01) and 1.33 ± 0.21 (*p* < 0.05) times more effective in protection of GSH depletion induced by HQ than BPA.

**Fig. 4:**
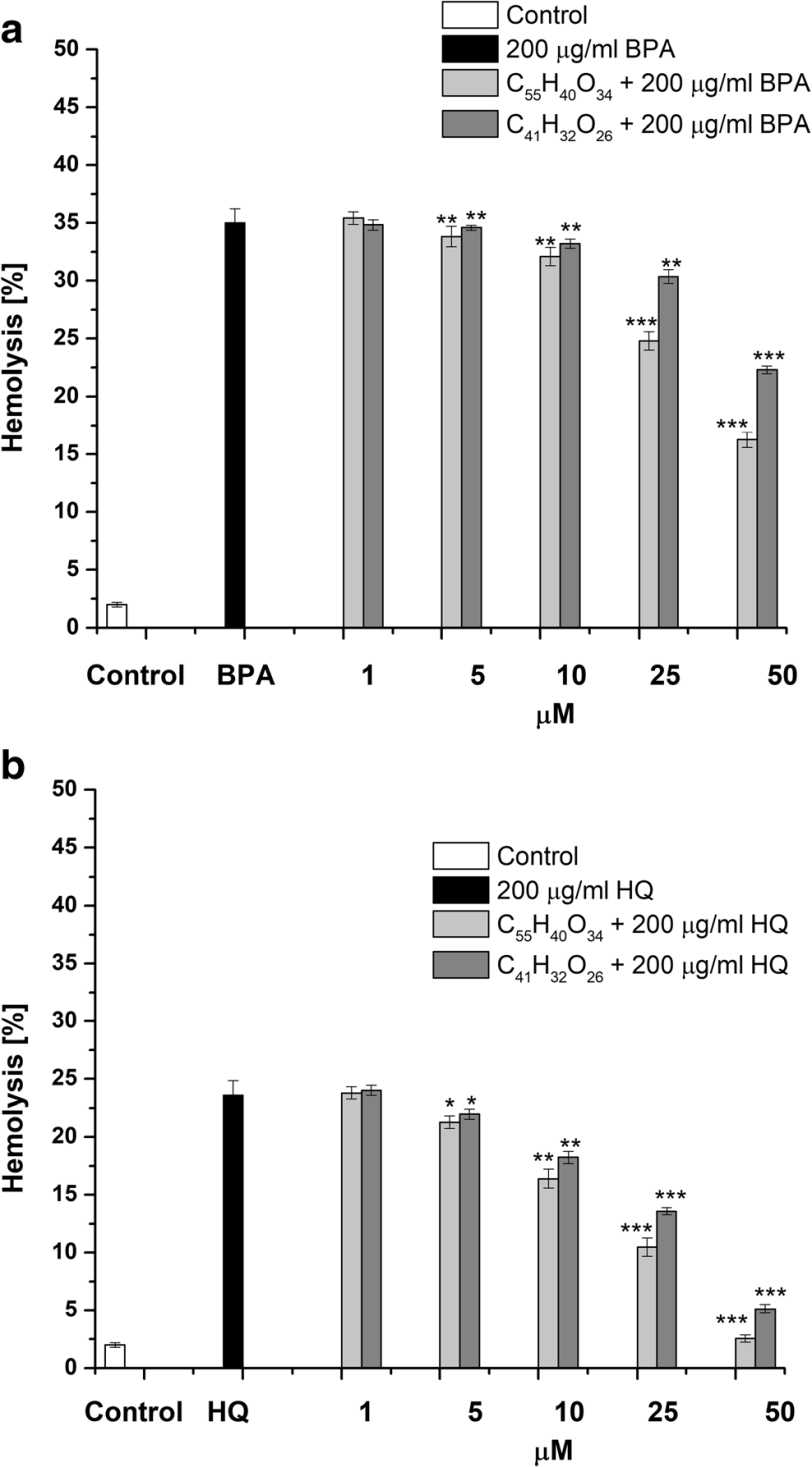
Protective effect of C_55_H_40_O_34_ and C_41_H_32_O_26_ against hemolysis induced by 200 μg/mL BPA (**a**) or HQ (**b**). The data are presented as means ± SE (*n* = 10). The effects of compounds were statistically significant according to one-way ANOVA test (*p < 0.05; **p < 0.01; ***p < 0.001)

We also tested the influence of BPA and HQ on erythrocyte integrity. As shown in Fig. 4, BPA and HQ at the concentration of 200 μg/mL caused 34.99 ± 2.25% (*p* < 0.001) and 23.58 ± 1.29% (*p* < 0.001) hemolysis of cells, respectively. A dose-dependent (10–50 μM) prior exposure to studied tannins statistically significantly reduced the hemolysis of erythrocytes with respect to cells treated only with BPA or HQ. In this case, C_55_H_40_O_34_ was found to protect the erythrocytes against hemolysis induced by BPA or HQ more effectively (1.56 ± 0.15 (*p* < 0.001) and 1.27 ± 0.13 (*p* < 0.01) times higher) as compared with C_41_H_32_O_26_.

It should be noted that as in the case of glutathione depletion, C_55_H_40_O_34_ and C_41_H_32_O_26_ exhibit 1.97 ± 0.18 (*p* < 0.001) and 2.4 ± 0.31 (*p* < 0.001) times higher protection from hemolysis of erythrocytes caused by HQ than BPA.

### The influence of C_55_H_40_O_34_ and C_41_H_32_O_26_ on the erythrocyte membrane fluidity

**Fig. 5:**
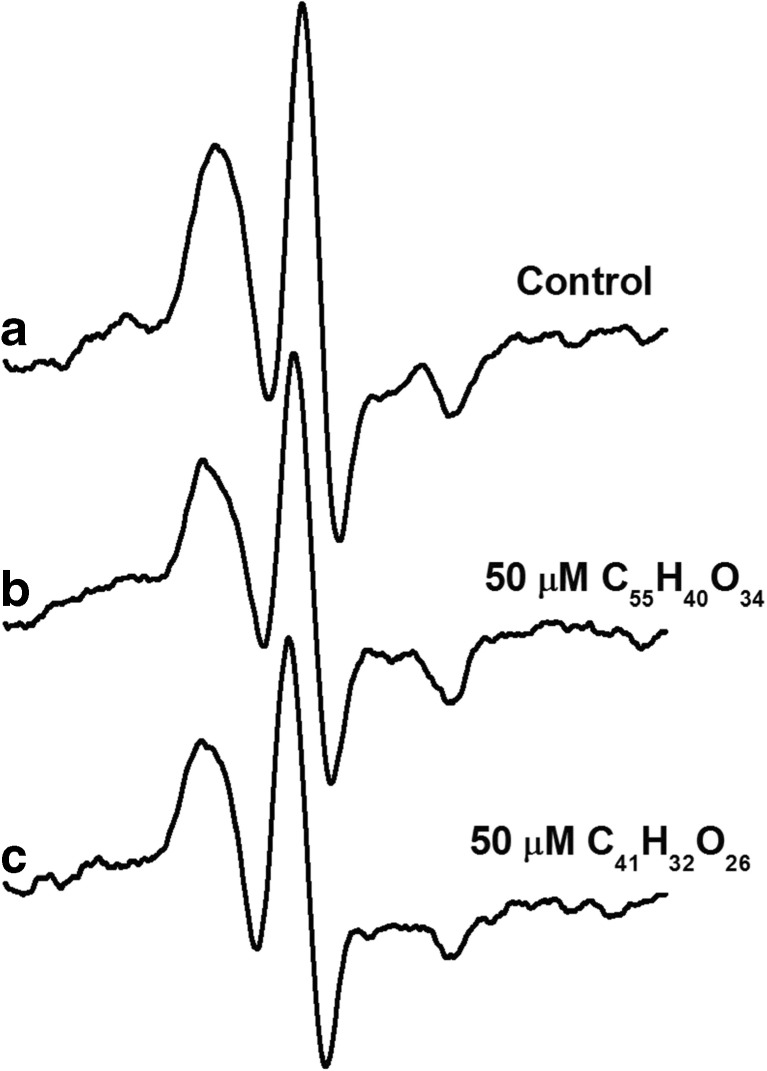
The EPR spectrum of erythrocytes labeled with 5-DS—control (**a**), C_55_H_40_O_34_ (**b**), and C_41_H_32_O_26_ (**c**)

We also examined, using 5-DS, the effect of C_55_H_40_O_34_ and C_41_H_32_O_26_ on the structure of erythrocyte membranes measured by EPR method (Fig. 5).

**Fig. 6:**
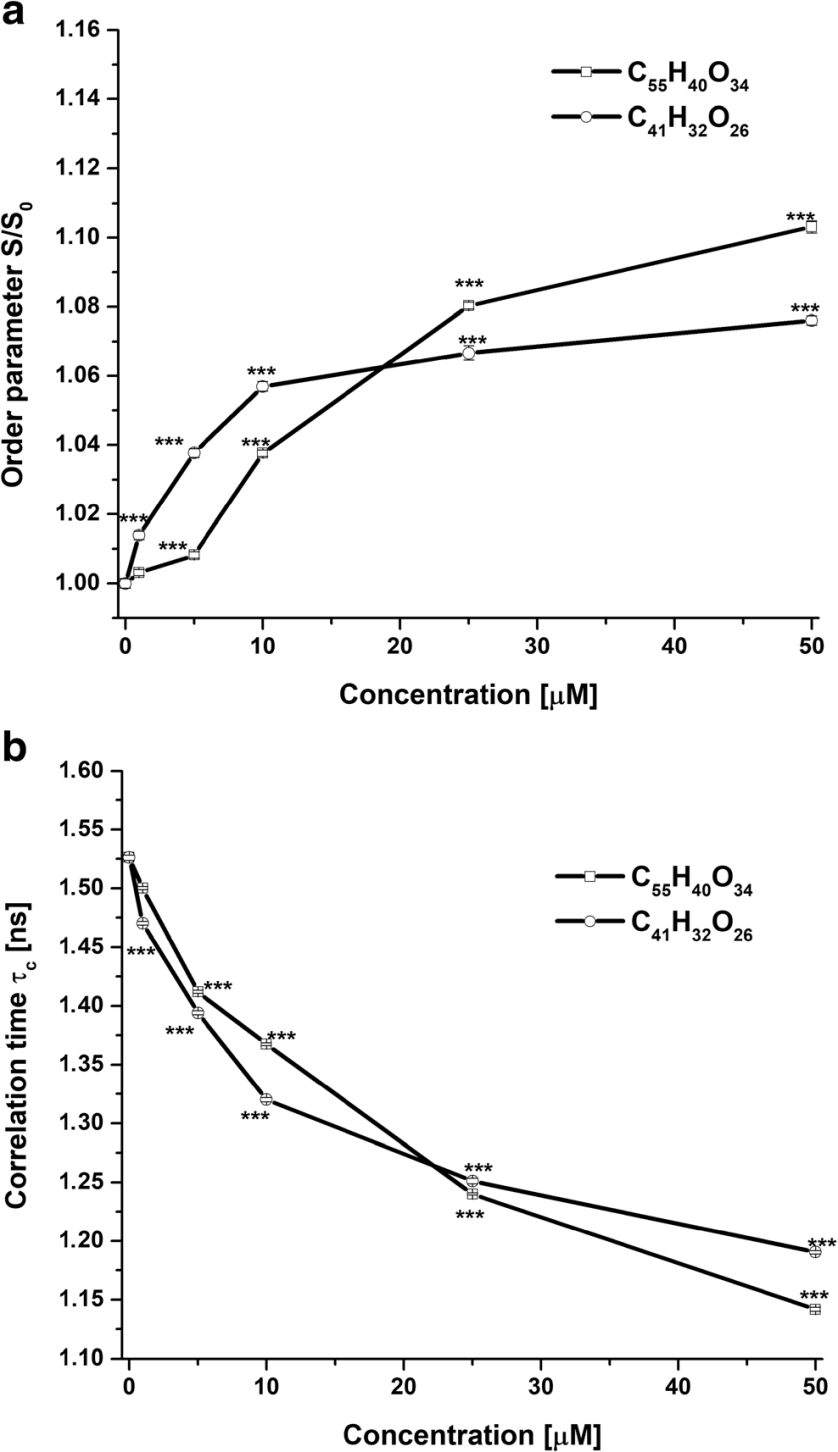
The effect of C_55_H_40_O_34_ and C_41_H_32_O_26_ on the order parameter *S*/*S*_0_ (**a**) and the rotational correlation time (*τ*_c_) (**b**) of the probe 5-DS in erythrocytes. The data presented are the means ± SE (*n* = 10). The effects of compounds were statistically significant according to one-way ANOVA test (*p < 0.05; **p < 0.01; ***p < 0.001)

The results are presented as the ratio (*S*/*S*_0_) between the order parameter in the presence (*S*) and in the absence (*S*_0_) of the studied tannins (Fig. 6a) and the rotational correlation time (*τ*_c_) (Fig. 6b).

C_55_H_40_O_34_ and C_41_H_32_O_26_ at a concentration of 50 μM statistically significantly increased the value of the order parameter *S*/*S*_0_ to 1.1031 ± 0.0017 (*p* < 0.001) and 1.076 ± 0.0013 (*p* < 0.001) in comparison with the control (without the addition of compounds taken as 1) (Fig. 6a) and also statistically significant decreased the *τ*_c_ to 1.1417 ± 0.0018 ns (*p* < 0.001) and 1.1903 ± 0.0015 ns (*p* < 0.001), respectively, in comparison with control 1.5259 ± 0.002 ns (Fig. 6b). The increase of the order parameter *S*/*S*_0_ and decrease the *τ*_c_ erythrocytes indicates that the tested compounds interacting with the erythrocyte membrane reduce the mobility of the probe in a region of localization of the 5-DS nitroxyl group that is associated with the increase in the membrane rigidity at the 5-carbon atom of hydrocarbon chain.

## Discussion

Increasing application of a variety of plastic products containing bisphenols in everyday life and accumulating information on its and its metabolites toxicity (Welshons et al. 2006, Konieczna et al. 2015; Halimi et al. 2016; Deb et al. 2016) lead to search of compounds which could prevent their negative influence on human organism. In the human body and, accordingly, in the blood, BPA and the products of its metabolism can get through drinking water, food and also dental material (Makarova et al. 2016; Terasaka et al. 2005; Nachman et al. 2014; Macczak et al. 2015, 2016). It means that a BPA and its metabolites can come into interaction with plasma proteins and hemocytes, and first of all with erythrocytes as carriers for xenobiotics. In this case the following options are possible: binding of the substance with membrane proteins, solubilization in lipids on cellular membranes and penetration of the substance into a cell and interaction with hemoglobin and other compounds.

Literature data point to the disturbance of the cell redox system resulting in oxidative cell damage as to one of the mechanisms of BPA and its metabolite HQ toxic effects (Terasaka et al. 2005; Michalowicz 2014; Wang et al. 2012; Peng et al. 2012, 2013). ROS formation due to the action of BPA was shown in a number of cells including mononuclear cells of human peripheral blood (Michalowicz et al. 2015), mouse lymphocytes (Lee and Lim 2010), hepatocytes (Hassan et al. 2012), hypothalamic neurons (Babu et al. 2013), and also erythrocytes (Macczak et al. 2015, 2016; Suthar et al. 2014; Verma and Sangai 2009). Cytotoxity of HQ connected with redox status was shown for human embryonic kidney cells (HEK293) (Shen et al. 2003), HL-60 cells (Terasaka et al. 2005), human lung alveolar epithelial cells (*A*_549_) (Peng et al. 2013), and erythrocytes (Sarkar et al. 2009).

Erythrocytes are very susceptible to oxidative stress due to the presence of high tension of oxygen, heme iron, and a high percentage of unsaturated fatty acids in the membrane. Oxidative stress in erythrocytes leads to eryptosis, a specific form of apoptosis, and as a consequence to the development of anemia (Macczak et al. 2016; Lang et al. 2012). In our experiments, we showed that BPA at the concentration of 200 μg/mL causes erythrocyte hemolysis and the formation of metHb. Our results are in line with those observed in other studies (Macczak et al. 2015; Suthar et al. 2014; Verma and Sangai 2009). It was suggested that the formation of metHb is a consequence of the oxidation of heme iron via superoxide anion formed by cells upon exposure to phenols (Macczak et al. 2015), while hemolysis may be the result of metHb aggregation and membrane lipid oxidation (Macczak et al. 2015; Suthar et al. 2014). The formation of ROS by BPA is assumed to occur via its oxidation to catechol with subsequent transformation of catechol into o-quinone. Even a small amount of BPA intermediate metabolic products may initiate oxidative stress in cells (Kovacic 2010). Another possible mechanism initiating oxidative stress may be a direct interaction of BPA with hemoglobin that was shown in model experiments by the methods of molecular docking. On the basis of these data, it was suggested that the binding of BPA with hemoglobin heme results in its dissociation and release of iron, which can induce the OH radical formation (Suthar et al. 2014).

We also demonstrated that BPA causes a decrease in the content of GSH in erythrocytes. This effect may be associated with the activation of glutathione-dependent peroxidases (GPx) that use GSH as a substrate in the reduction of lipid peroxides. The increased activity of this enzyme under treatment of BPA was shown in several studies (Wang et al. 2012, 2016).

A comparative study of the effect of HQ on the above parameters in erythrocytes showed that in the case of GSH and metHb, this BPA metabolite caused a stronger toxic effect than BPA itself. BPA at a concentration of 200 μg/mL increased the formation of metHb by 29.85 ± 2.48% (*p* < 0.001), compared with the control, whereas HQ at the same dose elevated it by 54.31 ± 1.91% (*p* < 0.001). HQ had a more pronounced effect on the level of the reduced GSH. Even at a concentration as low as 50 μg/mL, HQ caused a decrease in the level of reduced glutathione by 76.42 ± 2.81% (*p* < 0.001) while BPA at 200 μg/mL only by 41.56 ± 2.81% (*p* < 0.001). It should be noted that it was earlier shown that treatment of erythrocytes by HQ led to a decrease in the content of GSH and the formation of H_2_O_2_ but not metHb and LPO (Sarkar et al. 2009). It was demonstrated that the HQ-induced decrease of GSH occurred in the presence of the antioxidant butylated hydroxytoluene (BHT), which indicates rather direct formation of adducts with GSH. It was also shown previously, that the cytotoxicity of HQ, but not of BPA relative to HL-60 cells was blocked by *N*-acetyl-l-cysteine (NAC), a precursor of GSH. The toxicity of HQ was suggested to be mediated through the formation of thiyl radical derived from GSH (Terasaka et al. 2005) and HQ-GSH conjugates formation (Lau et al. 2010).

A decrease in the level of glutathione, the main redox buffer, as a result of HQ effect on erythrocytes, can also lead to an increase in the formation of methemoglobin, which was noted by us in the case of this toxicant.

We also showed that HQ caused hemolysis of erythrocytes and our data are consistent with the data of Sarkar et al. (2009). However, it should be noted that in this case, HQ at the same concentrations (200 μg/mL) produced less-pronounced effect in comparison with BPA (23.58 ± 1.29% vs. 34.99 ± 2.25%) (*p* < 0.01).

The reason for such a difference in effects is apparently to be found in different physicochemical properties of these toxicants and the ability to interact with membranes. The octanol-water partitioning coefficient for BPA was demonstrated to be equal to 4.32 while for HQ to 1.48 (Makarova et al. 2016), which means lower lipophilicity of HQ in comparison with BPA and a correspondingly weaker possibility of penetration into membranes.

In our study, we have also shown that despite some difference in the action of BPA and HQ tannins isolated from *Rhus typhina* L. exhibit protective against their cytotoxicity in erythrocytes. It was revealed that both investigated compounds inhibit hemolysis, the formation of metHb, and the increase in GSH content in the concentration dependent manner. The substance C_55_H_40_O_34_ containing more gallic acid residues and correspondingly a greater number of –OH groups as compared with C_41_H_32_O_26_ caused a significantly stronger protective effect. Inhibition of metHb formation and reduction of GSH content by the studied tannins may be the result of direct interaction of the compounds with ROS as well as their ability to chelate iron, which we showed previously (Olchowik et al. 2012; Olchowik-Grabarek et al. 2017).

We also studied influence of tannins on physical state of erythrocyte membrane by EPR method. The use of fatty acids with different position paramagnetic groups as EPR probes allows to monitors changes in membrane structure at various depth (Sonmez et al. 2013; Ajdzanovic et al. 2010, 2011, 2015; Mendanha et al. 2013; Gwozdzinski et al. 2011). We have chosen 5-DS because tannins with big molecular mass are known to affect the membrane surface without causing significant changes in the depth of the bilayer (Olchowik et al. 2012; Tarahovsky 2008).

We showed that both studied tannins caused an increase of the packing order parameter (*S*/*S*_0_) and decrease the *τ*_c_, which means that fluidity at the depth of 5th of carbon of fatty acid hydrocarbon chain in the erythrocyte membrane decreased. In this case, tannin with more gallic acid residues (C_55_H_40_O_34_) also has a greater effect as compared with C_41_H_32_O_26_.

However, it should be noted that there is not always a correlation between the number of aromatic rings and the OH groups in interaction with biopolymers. For example, it was shown that tannins containing 3 aromatic rings and 11 OH groups interact with albumin stronger than tannins containing 7 aromatic rings and 20 OH groups (Sekowski et al. 2014).

Increasing the degree of ordering of the membrane lipid organization may be viewed as the mechanism for preventing the erythrocyte swelling and hemoglobin release. It should be emphasized that in our earlier investigations, we showed that sumac tannins markedly depressed osmotic shock-induced hemolysis (Olchowik et al. 2012).

Erythrocyte membrane rigidification induced by studied tannins could also decrease the oxygen and toxicant penetration and inhibit oxidative process. The correlation between the membrane fluidity and spreading the free radical oxidation was revealed in many studies both for native membrane and liposomes (Olchowik et al. 2012; Soto-Arriza et al. 2008; Mosca et al. 2011; Strugala et al. 2016). Another possible mechanism for the protective effect of the studied tannins against oxidative stress in erythrocytes caused by BPA can be mediated by a direct neutralization of the toxicant via complex formation. This suggestion is based on the experiments where tannic acid was shown to form a complex with BPA with a high binding constant (Omoike and Brandt 2011). Perhaps in the case of the examined compounds, this mechanism is implemented, but this assumption requires further verification.

## Abbreviations

BPA: Bisphenol A
5-DS: 5-doxyl-stearic acid
EPR: Electron paramagnetic resonance
GSH: Glutathione
HQ: Hydroquinone
metHb: Methemoglobin
ROS: Reactive oxygen species
